# The Use of Osteogenon as an Adjunctive Treatment in Lower Leg Fractures

**DOI:** 10.3390/ph17111531

**Published:** 2024-11-14

**Authors:** Piotr Morasiewicz, Monika Zaborska, Michał Sobczak, Łukasz Tomczyk, Paweł Leyko, Andrzej Bobiński, Joanna Kochańska-Bieri, Daniele Pili, Krystian Kazubski

**Affiliations:** 1 Department of Orthopaedic and Trauma Surgery, Institute of Medical Sciences, University of Opole, Witosa 26, 45-401 Opole, Poland; 2 Faculty of Medicine, Institute of Medical Sciences, University of Opole, Witosa 26, 45-401 Opole, Poland; 3 Department of Food Quality and Safety Management, Faculty of Food Science and Nutrition, Poznan University of Life Sciences, Wojska Polskiego 28, 60-637 Poznan, Poland; 4 Bern Rehabilitation Center Heiligenschwendi, 3625 Heiligenschwendi, Switzerland; 5 Orthopedic and Trauma Department, G B. Mangioni Hospital, Via L. Da Vinci 49, 23900 Lecco, Italy

**Keywords:** ossein, hydroxyapatite, ossein–hydroxyapatite complex, tibial fractures, Ilizarov method

## Abstract

**Background:** The goal of the orthopedic treatment of fractures is to achieve bone union as rapidly as possible in the largest possible number of patients and to minimize the number of complications. The purpose of this study was to assess if the use of Osteogenon would have a positive effect on radiological and clinical parameters in patients with lower leg bone fractures treated with the Ilizarov method. **Methods:** We evaluated 26 patients who had their lower leg bone fractures treated with the Ilizarov method and received Osteogenon at our clinic in the years 2021–2023. The control group comprised 25 patients with lower leg bone fractures treated with the Ilizarov method who did not receive Osteogenon. We assessed the following parameters: time to achieving bone union, bone union rate, time to resuming normal physical activity, time to achieving pain relief, the number of patients reporting pain relief, and the rate of complications. **Results:** The median time to achieve bone union after lower leg bone fracture treated with the Ilizarov method was shorter in the Osteogenon group (108.5 days) compared to the control group (134 days), *p* < 0.001. Bone union was achieved in all the patients in the Osteogenon group and in 96% of the patients in the control group; the difference was not statistically significant. The median time to resuming normal physical activity was shorter in the Osteogenon group (22.5 weeks) compared to the control group (27 weeks), *p* < 0.001. The median time to achieving pain relief was shorter in the Osteogenon group (21 weeks) compared to the control group (30 weeks), *p* < 0.001. The proportion of patients who reported pain relief was 88.46% in the group receiving Osteogenon and 76% in the control group; this difference was not statistically significant. The number of complications was lower in the Osteogenon group (8 patients; 30.77%) compared to the control group (15 patients; 60%), *p* = 0.035. **Conclusions:** The use of Osteogenon has a beneficial impact on the treatment of lower leg bone fractures with the Ilizarov method. Osteogenon shortens the time to achieve bone union. Moreover, the use of the ossein–hydroxyapatite complex helps reduce the number of complications and shortens the time to achieve pain relief and to resume normal activities.

## 1. Introduction

The goal of the orthopedic treatment of all fractures, including tibia and fibula fractures, is to achieve bone union as rapidly as possible in the largest possible number of patients and to minimize the number of complications [1,2,3,4,5,6,7,8,9,10,11,12,13,14,15,16,17]. Some authors advocate the use of pharmaceutical agents as a means of improving bone tissue remodeling following fractures in order to achieve faster bone union [1,2,3,4,5]. Despite continual advances in the methods and instruments available for fracture reduction and bone fixation, some patients still experience delayed union or nonunion, which may be due to metabolic disorders, comorbidities, and societal aging [1,2,3,4,5,9]. Lower leg bone fractures, particularly multiple and compound ones and those located at the distal third of the lower leg, can result in problems with bone union (delayed union or nonunion [i.e., pseudoarthrosis]) more often than in other locations [3,6,7,8,9,10,11,12,13,14,15,16,17]. The Ilizarov method has been accepted worldwide for the treatment of tibia and fibula fractures, particularly compound and multiple fractures, or tibial pilon fractures [6,7,8,9,10,11,12,13,14,15,16,17].

The most important thing in fracture treatment, for orthopedists and patients alike, is to achieve strong bone union [1,2,3,4,5,6,10,12]. Successful fracture management involves early fixation removal and early rehabilitation, lowers the risk of limited range of motion and other complications, helps patients resume work and sports activities sooner, and reduces or eliminates pain [1,2,3,10,11,12]. In fracture treatment, it is important for doctors and patients to achieve the best possible treatment results, which allows for the fastest possible bone healing, achieving strong bone union and quick relief of pain, and return to work.

There are various medications intended to aid and accelerate bone union after fracture [1,2,3,18,19,20,21,22,23,24]. One such medication is an ossein–hydroxyapatite complex Osteogenon [1,2,3,18,19,20,21,22,23,24], which has been approved as a supplementary treatment for fractures and osteoporosis [1,2,3,18,19,20,21,22,23,24]. Osteogenon stimulates osteoblasts, inhibits osteoclasts, provides building materials for de novo bone growth, activates osteogenesis, stimulates bone metabolism, accelerates callus formation, and increases bone mass [1,2,3,18,19,20,21,22,23,24]. Ossein, the organic component of Osteogenon, contains type I collagen, beta-transforming growth factor, and insulin-like growth factors I and II, which stimulate osteoblast proliferation and osteogenesis [1,2,3,18,19,20,22,24]. The drug’s mineral component, hydroxyapatite, enters bone tissue and inhibits bone tissue resorption [2,3,19,20,22,24]. The organic and mineral components of Osteogenon and its potential mechanisms of action may have a positive effect on accelerating and increasing bone union, which, however, has not been fully researched and documented. The effect of Osteogenon on fracture healing has not been thoroughly investigated or described, and these effects have been described in only two publications so far [1,2]. This project aims to clearly identify the gaps in the existing research that it seeks to address. There have only been a handful of studies to assess fracture treatment with the use of Osteogenon. These studies were conducted on small study groups and were limited to evaluating only a few parameters [1,2]. There have been no studies assessing the effect of Osteogenon on the treatment of fractures with the Ilizarov method.

Fracture treatment with the Ilizarov method involves removing the fixator once the clinical and radiological evidence of union is established. The Ilizarov external fixator is removed immediately, or relatively soon after, the bone union is established. Conversely, other means of fracture fixation (plates and intramedullary nails) are removed a long time after the bone union is established or never. In light of the above, assessing the effects of medications on bone union in fractures treated with the Ilizarov method is more useful and effective than assessing the effects of medications on bone union with other fracture fixation methods.

We hypothesized that the use of Osteogenon would have a positive impact on tibia and fibula fracture treatment with the Ilizarov method.

The purpose of our study was to assess the effect the use of Osteogenon would have on radiological and clinical parameters in patients with lower leg bone fractures treated with the Ilizarov method.

The confirmation of the hypothesis of our study about the positive effect of Osteogenon on the treatment of fractures may allow for a more widespread use of Osteogenon in the treatment of fractures with various stabilization methods in many trauma centers.

## 2. Results

All the parameters evaluated in the experimental and control groups have been expressed in Table 1.

**Figure 1:**
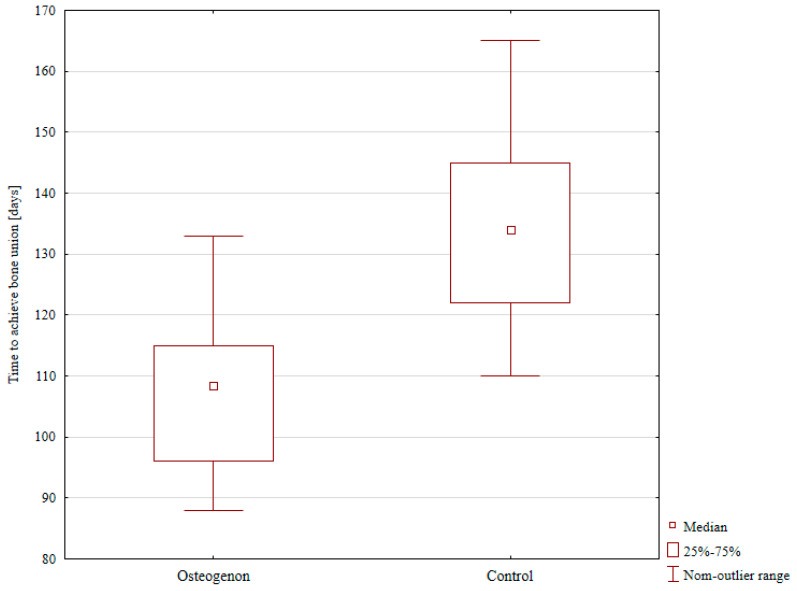
The median time to achieving bone union in the Osteogenon group and in the control group.

The median time to achieving bone union after lower leg bone fracture treated with the Ilizarov method was significantly shorter in the Osteogenon group (108.5 days) compared to the control group (134 days); *p* < 0.001, Table 1, Figure 1.

Bone union was achieved in all the patients in the Osteogenon group and in 96% of the patients in the control group, as shown in Table 1; the difference was not statistically significant.

The number of complications was lower in the Osteogenon group (8 patients; 30.77%) compared to the control group (15 patients; 60%), *p* = 0.035, as shown in Table 1. The following complications were observed in the Osteogenon group: Kirschner wire breakage requiring reoperation in one patient; limited range of motion at the ankle joint, which required intense exercise and rehabilitation, in five patients; and localized pin site infections, which subsided after oral antibiotic therapy, in two patients. Patients from the control group had one case of nonunion that required reoperation; one Kirschner wire breakage, which required reoperation; eight cases of limited range of motion at the ankle joint, which required intervention in the form of intense exercise and rehabilitation; and six cases of localized pin site infections, which resolved following oral antibiotic therapy.

**Figure 2:**
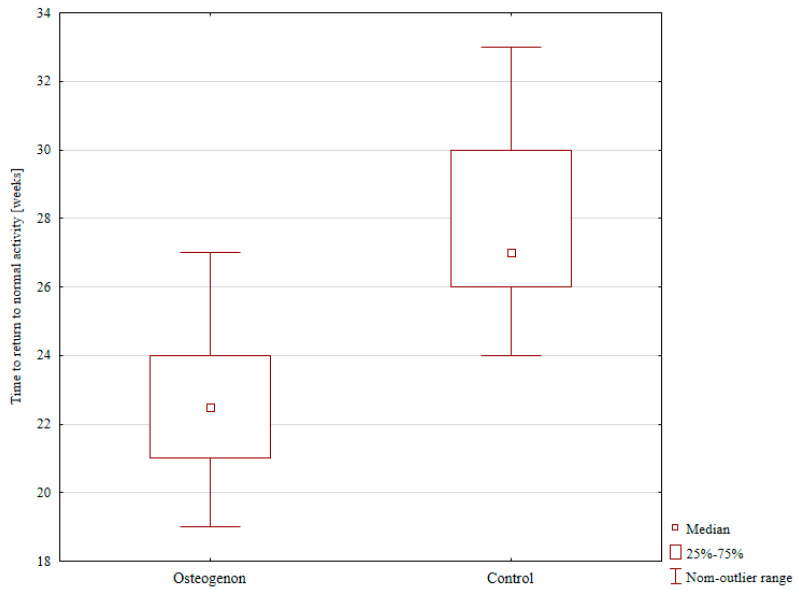
The median time to resuming normal physical activity in the Osteogenon group and in the control group.

The median time to resuming normal physical activity was 22.5 weeks in the Osteogenon group, and significantly longer (at 27 weeks) in the control group; *p* < 0.001, as shown in Table 1 and Figure 2.

**Figure 3:**
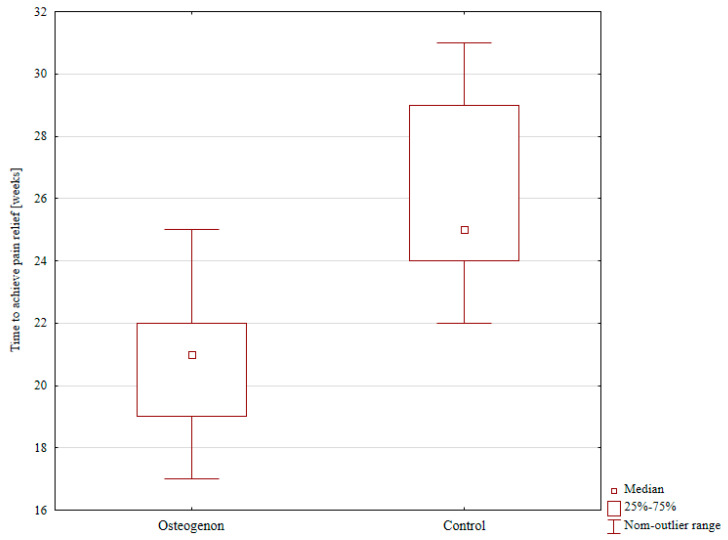
The median time to achieving pain relief in the Osteogenon group and in the control group.

The median time to achieving pain relief was 21 weeks in the Osteogenon group, and significantly longer at 30 weeks in the control group; *p* < 0.001, as shown in Table 1 and Figure 3.

The proportion of patients who reported pain relief was 88.46% in the group receiving Osteogenon and 76% in the control group; this difference was not statistically significant, as shown in Table 1.

## 3. Discussion

We assessed the effect of using Osteogenon on treatment outcomes in patients with lower leg bone fractures treated with the Ilizarov method. The patients receiving Osteogenon achieved bone union more rapidly, had lower complication rates, a shorter time to achieve pain relief, and a shorter time to resume normal physical activity in comparison with those in the control (no Osteogenon) group. The results of our study support our research hypothesis.

Various studies have shown the effectiveness of Osteogenon in slowing bone loss [1,3,18,20,22,23,24]. The use of Osteogenon increases bone mineral density in patients with osteoporosis [3,18,19,20,21,22,23,24]. Osteogenon stimulates bone metabolism and accelerates bone tissue formation [19,20,22,23,24].

Osteogenon components stimulate osteoblasts, inhibit osteoclasts, provide building materials for de novo bone growth, activate osteogenesis, stimulate bone metabolism, accelerate callus formation, inhibit bone tissue resorption, and increase bone mass [1,2,3,18,19,20,21,22,23,24]. Patients with fractures of the tibia and femur have been reported to have low bone mineral density; therefore, the use of Osteogenon may improve the process of fracture healing [3]. Osteogenon has been reported to increase bone tissue remodeling around femoral implants [1]. The ossein–hydroxyapatite complex improves bone mineral density and the quality of bone tissue [1,2,3,18,20,24]. Ossein, which is an organic component of the complex, stimulates osteoblast proliferation and the process of osteogenesis [1,2,3,20,24]. Osteogenon increases calcium levels and improves cortical bone parameters [1,3,24]. Osteogenon contains beta-transforming growth factors, and insulin-like growth factors I and II [1,2,3,18,19,20,22]. Studies assessing the pharmacokinetics and oral bioavailability of insulin-like growth factors I and II showed high plasma levels of these substances up to 48 h after oral administration (with the highest levels 4–8 h after administration), even in patients with renal impairment [25,26,27]. After oral administration, plasma levels of beta-transforming growth factor remain high for up to 24 h, with the highest levels at approximately 3 h after administration [28,29].

Twenty patients who received Osteogenon achieved radiological bone union 7–10 days earlier than those from the control group (without Osteogenon) [1]. Osteogenon has a positive effect on callus formation and normalizes the process of post-fracture bone remodeling [1,2,3].

Varga et al. observed fracture consolidation after 26 days in 15 radius fracture patients who received Osteogenon; this result was significantly better than that in the control group, not using Osteogenon [2]. The time to bone union in 42 patients receiving Osteogenon and treated for nonunion (pseudarthrosis) of the femur or tibia was 2–3 months shorter than that in the group not receiving Osteogenon [3].

Osteogenon activates post-fracture bone tissue formation and considerably shortens bone consolidation time [3,18]. Protein components of Osteogenon have mitogenic effects on osteocytes in vitro and improve bone tissue formation in vivo [20].

The mean time to achieve bone union in 76 patients with tibia fractures treated with the Ilizarov method was 148 days [6]. Foster et al. assessed 40 patients with tibia fractures treated with the Ilizarov method and observed bone union after a mean of 187 days [7]. Oztürkmen reported time to bone union in patients treated with the Ilizarov method ranging from 255 to 279 days depending on the exact location of the lower leg bone fracture [8]. Cibura et al. assessed 20 patients after tibia fracture treatment with an Ilizarov fixator and observed a mean time to bone union of 203 days [9]. A group of 30 patients with pilon fractures achieved a mean time to bone union of 154 days [10]. Wani reported bone union after a mean of 161 days [11]. McDonald et al. reported achieving bone union after a mean of 112 days [15]. In our study, the patients who received Osteogenon achieved bone union after a median of 108.5 days, which was a better result than those reported in the literature [6,7,8,9,10,11,15]. Our patients receiving Osteogenon achieved bone union faster than those from the control group, who did not receive Osteogenon. Our study results show a positive effect of Osteogenon on the rate of consolidation in the treatment of lower leg bone fractures with the Ilizarov method.

An earlier study showed that all the patients with tibia fractures treated with the Ilizarov method achieved bone union [6]. All the patients from the group assessed by Oztürkmen achieved bone union [8]. A group of 30 patients with pilon fractures achieved bone union [17]. Wani and Pal observed bone union in all the evaluated patients [11,12]. Foster et al. reported bone union in 90% of the patients treated with the Ilizarov method for tibia fracture [7]. Cibura reported bone union in 65% of the evaluated 20 patients with tibia fractures treated with the Ilizarov method [9]. McDonald et al. reported achieving bone union in 84% of the patients with pilon fractures treated with the Ilizarov method [15]. In our study, all the patients who received Osteogenon achieved bone union, which is a better result than those reported in the literature [6,7,8,9,10,11,12,15]. The Osteogenon group in our study achieved a higher bone union rate than the control group.

Rodianova et al. assessed the effect of Osteogenon in 20 patients with fractures treated via internal fixation at 3 and 12 months after surgery and observed pain relief in 63.3% and 86.7% of the patients using Osteogenon, respectively, and in 50% and 50% of the patients from the control group (not using Osteogenon), respectively [1]. Varga observed an absence of pain indicated on a visual analog scale (VAS) on day 35 by patients following radius fracture treatment with the use of Osteogenon [2]. Castelo-Branco et al. reported lower pain intensity at months 5–6 in 36 women taking Osteogenon for osteopenia than in those taking calcium carbonate [21]. Wani et al. observed persistent pain in 25% of the patients after tibia fracture treatment with the Ilizarov method [11]. Pain at the fracture site after surgery with the Ilizarov method was also indicated by 43.26% of the patients who were evaluated by Jeremica [14]. The proportion of patients with pain relief in our study was greater than those reported in the literature [1,11,14]. Our Osteogenon group and control group (without Osteogenon) exhibited comparable rates of pain relief.

The shorter duration of pain following surgical treatment observed in our study in the group of patients taking Osteogenon than in the control group may have been due to several factors. According to some authors, Osteogenon has analgesic properties [1,2,3,4,7]; however, this analgesic effect of the drug is not fully understood [1,3,7]. One of the possible mechanisms of action of Osteogenon may be associated with its inhibition of osteoclasts, which release pain mediators [7]. Another theory of the drug’s analgesic effect states that the process of pain perception is associated with growth factors, and Osteogenon contains growth factors among its components [7]. Rodianova reported a shorter duration of pain in fracture patients who underwent internal fixation and took Osteogenon in comparison with that in patients who did not take Osteogenon [1]. Osteogenon helps achieve bone union more rapidly, which limits and eliminates bone fragment movement that might cause pain.

Five percent of patients with lower leg bone fractures treated with the Ilizarov method and evaluated by May et al. developed complications (malunion) [6]. Kirschner wire breakage was reported in 5% of the patients assessed by Foster et al. [7]. Complications in the form of deformities were observed in 8.3% of the fracture patients treated with the Ilizarov method [8]. Cibura et al. evaluated 20 patients with tibia fractures treated with an Ilizarov fixator and reported nonunion in 35% of the study group [9]. Complications were observed in 13.33% of the patients with pilon fractures treated with the Ilizarov method [10]. Wani et al. reported post-treatment limb shortening of >1 cm in 3.33% of the patients with tibia fractures treated with the Ilizarov method [11]. In a group of patients treated with the Ilizarov method and assessed by Pal et al., the fixation became destabilized in 6.25% of the patients, pin breakage occurred in 6.25% of the patients, and delayed union in 18.75% [12]. Kumar et al. reported delayed union in 25.4% of their study group [23]. Other authors reported nonunion in 16% of the patients with pilon fractures treated with the Ilizarov method [25]. In our study, 30.77% of the patients who took Osteogenon developed complications, which is only a slightly worse result than those reported in the literature [6,7,8,9,10,11,12,13,15]. Our patients with tibia fractures treated with the Ilizarov method who were receiving Osteogenon developed fewer complications than those from the control group (without Osteogenon).

A study of 20 fracture patients who underwent external fixation showed that those who received Osteogenon had a shorter rehabilitation period and resumed their normal activities sooner than those from the control group (without Osteogenon) [1]. The resumption of their normal activities was possible 3–5 months after fracture fixation [1]. The patients in our study resumed their normal activities after a period similar to those reported in the literature [1]. The Osteogenon group resumed their normal activities significantly sooner than the control group. This may be due to the fact that the patients from the Osteogenon group achieved bone union more rapidly, had their Ilizarov fixator removed earlier, reported pain relief sooner, and developed fewer complications than those from the control group. All these factors may have affected the patients’ ability to resume their normal activities earlier. A systematic review found that vitamin D alone had little effect on fracture healing, slightly increased union rates, and modestly improved functional outcomes [30]. Due to the above, our results encourage the use of Osteogenon as an adjuvant in the treatment of fractures.

Varga et al. reported no side effects of Osteogenon in 15 fracture patients [2]. In another study, Osteogenon was well tolerated, with side effects reported in 3.2% of the patients [18]. Castelo-Branco observed side effects following the use of Osteogenon in only 0.3% of the patients [20]. In our experimental group, there were no side effects after Osteogenon use, which indicates that the drug is well tolerated by patients with lower leg bone fractures treated with the Ilizarov method.

Osteogenon has limitations in use; it is not a drug that can be used by every patient. Osteogenon is registered for the treatment of osteoporosis and as an adjunctive therapy in the treatment of fractures. This medicine is intended for adults. Contraindications to the use of Osteogenon include severe renal failure, dialysis, hypercalcemia, hypercalciuria, calcium nephrolithiasis or tissue calcification, and age < 18 years. The administration of Osteogenon in combination with vitamin D should be closely monitored by checking the concentration of calcium in the blood serum and urine. There are no publications in the available literature assessing the long-term results of the use of Osteogenon on bone union after fractures.

One of the limitations of our study is a relatively small sample size, which was a result of our desire to assess a homogeneous group of patients treated with the Ilizarov method and the relatively low incidence of cases with indications for this method of treatment. The relevant literature contains papers from studies assessing the effect of Osteogenon on treatment in study groups that are similar in size to, if not smaller than, ours [1,2]. Another limitation of our study is its retrospective nature, which is due to our intention of using the available extensive medical and radiological records. Other studies assessing the effect of Osteogenon on fracture treatment have also been retrospective in nature [1,2,3]. The relatively small number of study participants and the retrospective nature of the study may have an impact on possible effects on the generalizability of the results.

The strengths of our study include an identical treatment protocol, including the surgery technique for lower leg bone fractures with the use of the Ilizarov method in all the patients from both the Osteogenon and control group, all the surgical procedures being performed by the same orthopedic surgeon, an identical Osteogenon regimen in the experimental group, an identical rehabilitation protocol for all the patients, and an identical schedule of radiological and outpatient follow-up visits for all the patients. One strength of our study is the fact that the experimental and control groups did not differ significantly in terms of the proportion of open fractures, diabetes, or smokers.

In the future, we are planning to conduct similar studies in larger groups of patients and to conduct a prospective study to assess the effect of Osteogenon on fracture treatment with the Ilizarov method. We plan to perform a similar study with a longer observation period and assess the effect of Osteogenon on the treatment of nonunions using the Ilizarov method and bone union after Osteotomies with the Ilizarov method.

## 4. Material and Methods

We evaluated 26 patients who had their lower leg bone fractures treated with the Ilizarov method and took Osteogenon at our clinic in the years 2021–2023. Osteogenon is a complex of organic ossein and inorganic hydroxyapatite. Each tablet contains 178 mg of calcium, 82 mg of phosphorus, type I collagen, beta-transforming growth factor, and insulin-like growth factors I and II [1,2,3,18,19,20,22].

The patients were included in the study based on their history of lower leg bone fracture treatment with the Ilizarov method, receiving Osteogenon throughout their treatment, complete medical records, complete radiological methods, not receiving any other medications that might affect bone tissue remodeling, and a follow-up period of 12 months after treatment completion. The study exclusion criteria were incomplete medical records, incomplete radiological records, receiving medications that might affect bone tissue remodeling, a follow-up period of less than 12 months after treatment completion, fracture with bone fragment loss, and/or the use of a bone transport technique.

The patients received two Osteogenon tablets daily throughout the entire period of fracture treatment, from the time the Ilizarov fixator was mounted until the time it was removed.

Due to the retrospective nature of the study and the fact that the evaluated drug had already been approved and used in clinical practice, the study did not require an ethics committee’s approval according to Polish regulations in force at the time the study began.

All the surgical procedures with the use of the Ilizarov method in the experimental and control group were performed by the same experienced orthopedic surgeon. After the inclusion and exclusion criteria were applied, a total of 26 patients (6 females and 20 males), aged 20 to 68 (mean age 45 years), all of whom took Osteogenon, were included in this study.

The control group comprised 25 patients with lower leg bone fractures treated with the Ilizarov method, who did not receive Osteogenon or other drugs that might affect bone union. All the patients from the control group were operated on by one orthopedic surgeon—the same one who operated on the patients from the experimental group taking Osteogenon. The patients from the control group received treatment at one center in the period between 2017 and 2020, at a time when the treatment protocol did not include Osteogenon or any other medications that might affect bone union. The control group comprised 25 patients (6 females and 19 males) matched by age, BMI, and sex to those from the experimental group, as shown in Table 2.

The study groups were comparable in terms of the rates of smokers, peripheral vascular disease, diabetes, open fractures, and Gustilo–Anderson fracture types, as shown in Table 2. There were no patients with osteoporosis, bone fragment loss, or limb shortening either in the experimental (Osteogenon) or control group. The patients in both groups underwent only fracture reduction and external fixation with the Ilizarov method without bone transport.

The sample size resulted from the broad and restrictive criteria for inclusion and exclusion in the study, the possibility of selecting a homogeneous and similar control group, and the number of fractures and indications for the use of the Ilizarov fixator of lower leg fractures in a limited group of patients (mainly open fractures and comminuted fractures). The retrospective nature of the study resulted from the desire to quickly present, for many recipients, the significant positive effects of the use of Osteogenon in the treatment of fractures using the Ilizarov method, while a prospective study requires a longer time.

Radiological and clinical assessments were performed every 3–6 weeks from the time the Ilizarov fixator was mounted to the time bone union was observed and the fixator removed. Following fixator removal, radiological and clinical assessments were conducted every 6 weeks. Bone union was determined based on radiological and clinical evidence [11,15,17]. The radiological union was confirmed by the presence of at least 3 out of 4 cortices and the trabecular bridging of the fracture gap [11,15,17]. The clinical bone union was determined in patients with no pain or pathological mobility of bone fragments following an attempt to forcibly move the bone fragments after loosening the fixator and having the patient walk without feeling any pain while bearing weight on the injured limb [11,15,17]. Once the clinical and radiological parameters of the bone union were established, the Ilizarov fixator was loosened. During the subsequent 7 days, the patients were allowed to walk while bearing weight on the injured limb. If a follow-up radiograph performed 7 days later showed no secondary displacement of bone fragments, the external fixator was removed. Afterwards, the patients were advised to walk with two crutches while gradually increasing weight-bearing on the injured limb over 3–6 weeks. All the patients had an identical rehabilitation protocol.

This study assessed the following clinical and radiological parameters: time to achieving bone union, bone union rate, time to resuming normal physical activity, time to achieving pain relief, the number of patients reporting pain relief, and the rate of complications.

Time to achieving bone union was defined as the number of days from the Ilizarov fixator being mounted to it being removed.

The bone union rate—expressed as a percentage—was defined as the proportion of patients who achieved clinical and radiological bone union.

Time to resuming normal physical activity was defined as the time—expressed in weeks—from the Ilizarov fixator being mounted to the patient returning to work or school.

Time to achieving pain relief was defined as the period of time—expressed in weeks—from the Ilizarov fixator being mounted to pain medication being discontinued.

The number of patients reporting pain relief was defined as the proportion of patients—expressed as a percentage—who were not on any painkillers at long-term follow-up.

Complications were assessed based on medical and radiological records. The following complications were considered: nonunion, delayed union, destabilization of the fixation, reoperation, implant breakage, pain, edema, limited range of motion, vascular damage, nerve damage, and infections.

All these parameters were assessed based on each patient’s complete medical and radiographic records. Subsequently, the results observed in the experimental (i.e., Osteogenon) group were compared with those of the control group.

### Statistical Analysis

Data were statistically analyzed using Statistica 13.1. The Shapiro–Wilk test was used to check for the normality of distribution. The Mann–Whitney U test was used to compare the quantitative variables. In the case of qualitative variables, the Pearson chi-square test was used. The significance level was set at *p* < 0.05.

## 5. Conclusions

The use of Osteogenon has a beneficial impact on the treatment of lower leg bone fractures with the Ilizarov method.

Osteogenon significantly shortens the time to achieving bone union and significantly shortens the time the external fixator stays in place for treating lower leg bone fractures with the Ilizarov method.

The use of the ossein–hydroxyapatite complex helps significantly reduce the number of complications and significantly shortens the time to achieving pain relief and to resuming normal activities in patients after tibia and fibula fractures treated with the Ilizarov method in comparison with patients who do not use this medication.

## Figures and Tables

**Table 1:** Selected parameters in the Osteogenon group and control group.

Analyzed Variable		Group		Z	*p*-Value	
		Osteogenon Group [*n* = 26]	Control [*n* = 25]			
	Value					
Time to achieve bone union [days]	Q1	96	122	−5.080	<0.001	
	Median	108.5	134	−4.89898		
	Q3	115	145	−5.07798		
Time to return to normal activity [weeks]	Q1	21	26	−4.898	<0.001	
	Median	22.5	27	−4.89898		
	Q3	24	30	−5.07798		
Time to achieve pain relief [weeks]	Q1	19	24	−5.099	<0.001	
	Median	21	25	−4.89898		
	Q3	22	29	−5.07798		
				χ^2^	df	*p*-value
Pain relief	% of observations	88.6	76	1.36	1	0.243
Bone union		100	96	1.06	1	0.303
Complications		30.77	60	4.46	1	0.035

Z—standardized value of the Mann–Whitney test; χ^2^—value of the chi-square test statistic; df—degrees of freedom; p-value for the Mann–Whitney U or chi-square test; Q1, Q3—the 1st and 3rd quartiles.

**Table 2:** Detailed characteristics of the study and control groups.

Analyzed Variable		Group		Z	*p*	
		Osteogenon Group [*n* = 26]	Control [*n* = 25]			
	Value					
Age [years]	Q1	37	23	0.028	0.977	
	Median	44.5	44	−4.89898		
	Q3	54	27	−5.07798		
BMI [kg/m^2^]	Q1	23	23	0.009	0.992	
	Median	26	26	−4.89898		
	Q3	28	27	−5.07798		
				χ^2^	df	*p*
Male	% of observations	76.92	76	0.006	1	0.938
Smoker		38.46	40	0.012	1	0.91
Diabetes mellitus		11.54	12	0.002	1	0.959
Peripheral vascular disease		15.38	16	0.003	1	0.951
Open fracture		50	48	0.02	1	0.886
Gustilo–Anderson II		34.62	36	0.01	1	0.917
Gustilo–Anderson IIIA		11.54	8	0.18	1	0.67098
Gustilo–Anderson IIIB		3.85	4	0.001	1	0.977

Z—standardized value of the Mann–Whitney test; χ^2^—value of the chi-square test statistic; df—degrees of freedom; *p*-value for the Mann–Whitney U test or chi-square; Q1, Q3—1st and 3rd quartiles.

## Data Availability

The data presented in this study are available upon request from the corresponding author.
